# N-doped Carbon Coated CoO Nanowire Arrays Derived from Zeolitic Imidazolate Framework-67 as Binder-free Anodes for High-performance Lithium Storage

**DOI:** 10.1038/s41598-019-42371-y

**Published:** 2019-04-11

**Authors:** Dongxia Wang, Bo Yan, Yujuan Guo, Long Chen, Feng Yu, Gang Wang

**Affiliations:** 1 School of Chemistry and Chemical Engineering, Key Laboratory for Green Processing of Chemical Engineering of Xinjiang Bingtuan, Shihezi University, Shihezi, 832003 China; 2 Key Laboratory of Materials-Oriented Chemical Engineering of Xinjiang Uygur Autonomous Region, Shihezi, 832003 China

## Abstract

To realize large lithium storage capacity and excellent rate capability lithium ion batteries, highly electrochemically active materials and rational design of structure are desirable. Here, we successfully synthesized CoO@N-doped carbon nanowire arrays derived from zeolitic imidazolate frameworks-67 (ZIF-67) on Ni foam (denoted as CoO@N-C/NF). Each CoO@N-C nanowire was built up of numerous ordered *in-situ* nitrogen-doped carbon coated CoO nanoparticles (around 20 nm) after annealing treatment. Benefited from the unique structural features, when served as anode for lithium ion batteries, the CoO@N-C/NF exhibit superior initial Coulombic efficiency of 78.04%, and excellent electrochemical cyclability (1884.1 mAh g^−1^ at 1 A g^−1^ after 100 cycles) and good rate capability (1169.2 mAh g^−1^ at the rate of 5000 mA g^−1^). To our knowledge, this is the highest capacity with similar electric current density that has been reported for CoO-based materials. Our results indicate that the CoO@N-C/NF electrode without any auxiliary materials are expected to open up new opportunities for CoO-based material to power electronic devices.

## Introduction

As a major energy storage device, lithium ion batteries (LIBs) are the most prevailing power sources for high-end and portable electronic products, such as laptops and mobile phones for quite a few years^1–5^. Nevertheless, the current commercial graphite anode, hosts a low specific capacity (372 mAh g^−1^), far from our expectations. Therefore, there is an urgent need for extensive exploration and special design of new electrode materials in order to improve the specific capacity^6–8^. In the various anode materials, transition metal oxides usually possess high theoretical capacity in view of the conversion mechanism (MO_x_ + 2xLi + 2xe^−^ ↔ M + xLi_2_O) by transmitting a number of electrons^9^. Since the above discovery, CoO has been regarded as a promising substitute to graphite due to its relatively high lithium storage capacity (716 mAh g^−1^). However, CoO LIBs anodes suffer from a large specific volume change during discharge-charge cycles and low conductivity, which often results in performance degradation. Thus, rational modification and design are needed for CoO-based electrodes material with high reversibility and excellent rate performances.

Great endeavors have been made to optimize the electrochemical properties of CoO-based electrodes. A general strategy is to design nanostructured materials with hierarchical architecture^10^. Among the previous works, one-dimensional(1D) nanomaterials with hierarchical structures have been extensively developed and proved to be the optimal structure of electrochemical electrodes due to their better electron transport capacity, higher surface-to-volume ratios, and relatively lower volume change during the lithiation/delithiation processes compared with other higher dimensional nanomaterials^11–13^. However, these 1D nanomaterials usually need to be mixed with polymeric binder and carbon black, and further pressed onto current collector. The addition of conductive additive and binder inevitably sacrifices overall energy storage capacity and decrease the electrical conductivity of the electrode materials, thus hindering their potential application in high-performance energy storage devices^14–16^. Alternatively, the fabrication of integrated adhesive-free electrodes by directly growing metal oxide nanowire arrays on self-supporting three-dimensional (3D) porous conductive substrates have received tremendous attention. The integrated electrode avoids the use of any adhesive or conductive additives, provides a larger electrode/electrolyte contact area, and provides a 3D interconnected network path for electrons and ions to produce effective reaction kinetics during charge/discharge. For example, Liu *et al*.^17^ synthesized mesoporous CoO nanowires on Ni foam through a hydrothermal reaction, which shows a reversible specific capacity of 1398 mAh g^−1^ after 80 cycles. Jiang *et al*.^18^ reported porous CoO nanowire arrays on Ti foil, which exhibits good high-rate capability. In spite of the above reports, the electrochemical performance of CoO has been improved to a certain extent, but its intrinsic poor electrochemical conductivity and dramatic volume variation in the cycle process still greatly inhibited its long cycling stability and superior rate performance.

Nitrogen-doped carbon coated metal oxide nanowires with a unique core-shell structure have proven to be an effective strategy for solving the above problems^19,20^. The N-C shell can not only accommodate a large volume expansion/contraction, but also effectively increase the conductivity of the metal oxide. Many reports have certified that 1D core-shell structured nanomaterials exhibit superior performance when used as anode materials for LIBs^21,22^. However, the process of forming a high quality N-C shell is relatively difficult and complicated. More recently, Gan *et al*.^23^ synthesized ZnO/NC-Z nanorods adopted a strategy for ZnO nanorods *in-situ* encapsulated by ZIF-8. This distinctive ZnO/NC-Z nanorods electrode has excellent electrochemical properties. Nevertheless, there has been no report on *in-situ* coated CoO nanowire arrays with nitrogen-doped carbon derived from MOFs.

Herein, we demonstrate a simple and extensible strategy to obtain integrated and binder-free CoO@N-doped carbon nanowire arrays derived from zeolitic imidazolate framework-67 (ZIF-67) on Ni foam (denoted as CoO@N-C/NF), in which each nanowire composed of ordered CoO nanoparticles encapsulated by nitrogen-doped carbon. With such a unique electrode design, the advantages can be listed as follows. Firstly, Ni foam with three-dimensional structure was adopted as the collector instead of traditionnal two-dimensional conductive collectors, due to its outstanding mechanical stability, higher electron conductivity, better corrosion resistance, and low cost. Secondly, the CoO@N-C nanowire arrays was grown directly on Ni foam, without requiring the addition of binders and thus avoiding the loss of lithium storage capacity. Thirdly, the unique 1D porous nanostructures provides a larger surface-to-volume ratio, shorten the distance of Li^+^ and charge diffusion and facile strain accommodation in the process of lithiation reaction, which is conducive to enhance the lithium storage properties. Fourthly, the outer N-doped carbon can not only promote the ion/electron transport kinetics, but also decrease the energy barrier of ion migration during charge/discharge processes. Benefited from the the advantages listed above for lithium storage, the designed CoO@N-C/NF exhibited excellent lithium-storage capacity and good rate capability as anode materials for LIBs.

## Results and Discussion

**Figure 1:**
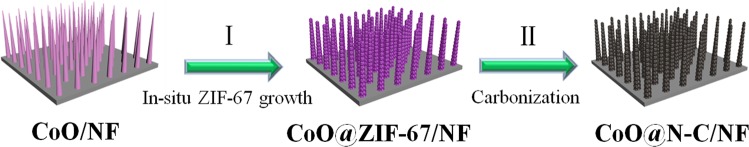
The above illustration shows that the synthesis procedures of CoO@N-C/NF.

**Figure 2:**
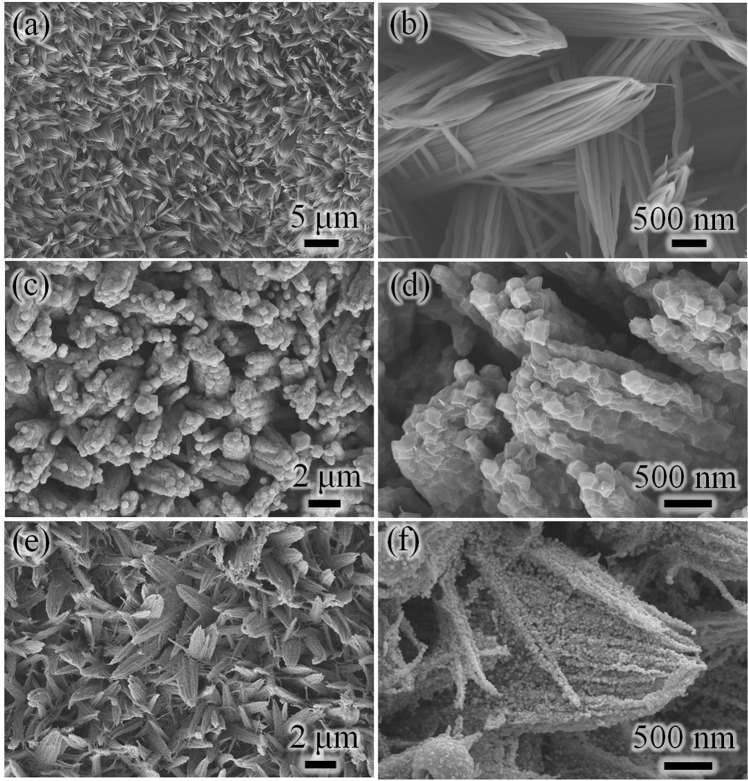
SEM image of (**a**,**b**) as-prepared CoO/NF; (**c**,**d**) CoO@ZIF-67/NF; (**e**,**f**) CoO@N-C/NF.

The synthetic strategy of the integrated CoO@N-C/NF has been displayed in Fig. 1. Initially, a piece of cleaned Ni foam preprocessed was put into the homogeneous solution of Co^2+^ to conduct a hydrothermal reaction and then annealing to obtain the CoO/NF^24^. After the annealing, the Ni foam was uniformly covered with the vertical CoO nanowire arrays with diameter of 50 nm and length of 1.5–2.5 μm (Fig. 2a,b). CoO@ZIF-67/NF were prepared by using CoO/NF as sacrificial templates in the component solvent of absolute ethyl alcohol, H_2_O and Hmim. During the chemical transformation, with the help of solvent, CoO nanowires can dissolve itself into Co^2+^ ions, while Hmim acted as the ligand and etching reagent. As seen from the images of morphologies (Fig. 2c,d), it is obvious that the uniform CoO@ZIF-67 nanowire arrays were achieved after the ion exchange process, well adhering to the Ni foam substrate.

During the morphology evolution of CoO@ZIF-67/NF, ZIF-67 subunits are gradually grown on the surface of CoO/NF (Fig. S1). It is worth mentioning that the reaction time plays a significant role on the of the thickness of the ZIF-67 shell. At the beginning of the reaction (5 min), only some small ZIF-67 particles are formed on the surface of the CoO/NF (Fig. S1a,d). With the prolongation of reaction time(1 h), more nanoparticles were stacked up to form a continuous layer (Fig. S1b,e). The thickness of the ZIF-67 shell increased to ~50 nm (Fig. S1c,f) when the reaction time was 6 h. However, the excess of time will lead to the overgrowth and severely agglomerate of polyhedral subunits (Fig. S2).

Finally, the well-aligned CoO@ZIF-67/NF were subsequently annealed under N_2_ gas and air in turn. SEM images (Fig. 2e,f) show that the unique shape of the CoO@N-C nanowires is similar to that of an ear of wheat-like nanostructure. Compared with the pure CoO nanowire, each nanowire of target product with a hierarchical morphology, which assembled by many nanoparticles  ≈20 nm in size. The introduction of carbon layer supress the agglomeration of the CoO nanoparticles, in addition, the direct connection between CoO@N-C nanowires and the growth substrate, avoiding the binder could greatly improve transmission rate of Li ions and electrons into/out of the CoO@N-C nanowires architecture.

**Figure 3:**
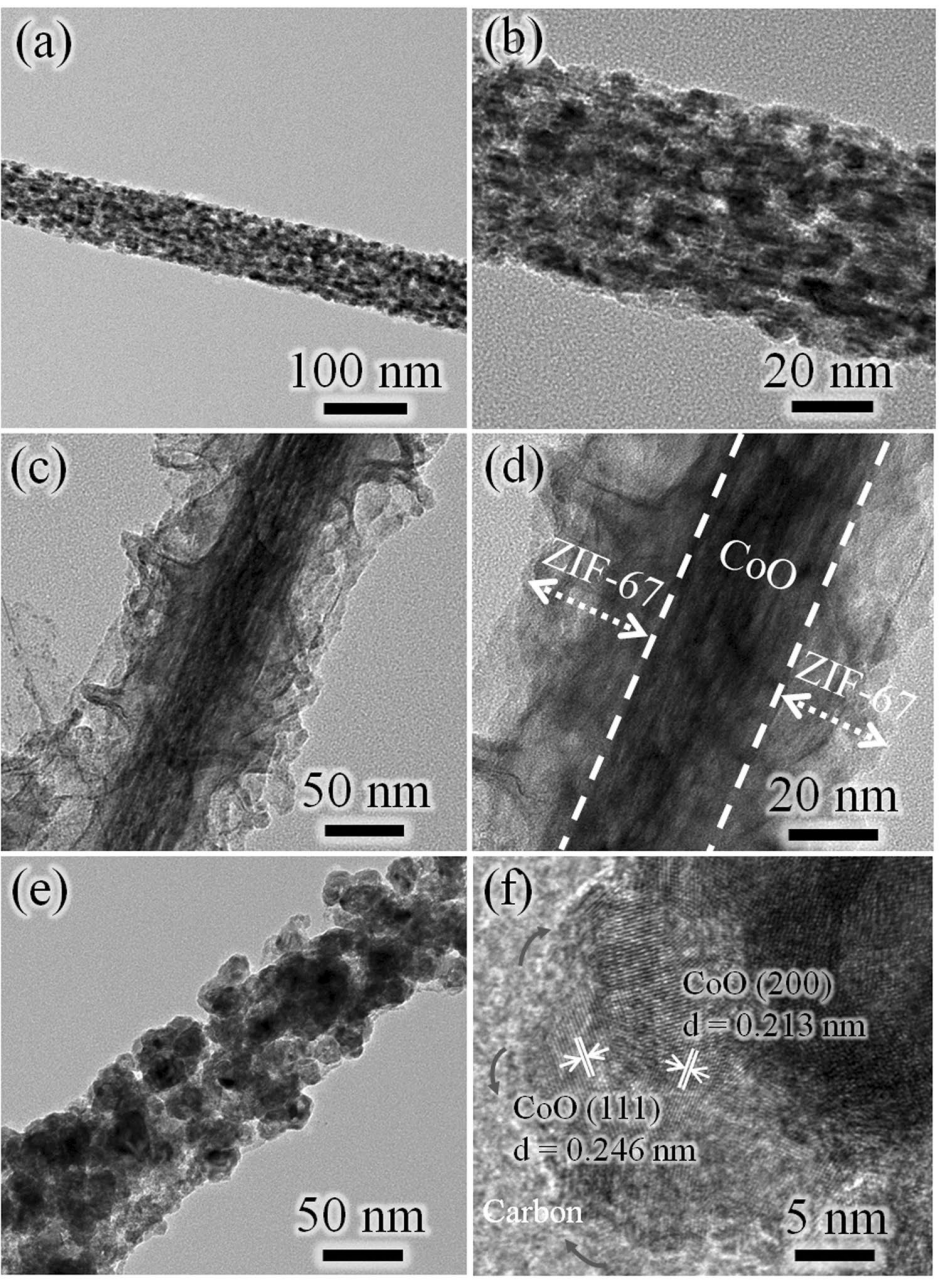
Low and high resolution TEM images of: (**a**,**b**) CoO nanowire; (**c**,**d**) CoO@ZIF-67 core-shell nanowire; (**e**,**f**) core-shell CoO@N-C.

TEM results provide more details about the microstructure and morphology of CoO nanowire precursors and products. Typical TEM image (Fig. 3a) shows the single CoO nanowire with an average width of 70 nm. Obviously, there are many pore detects on the surface of CoO nanowires, due to the release and decomposition of gases(CO_2_, H_2_O) during annealing processes (Fig. 3b). Figure 3c shows the CoO@ZIF-67 nanowire with a typical core-shell configuration. According to the high magnification TEM (Fig. 3d), the shell thickness of ZIF-67 with light contrast was approximate 60 nm, while the core CoO nanowire with dark contrast was much thinner in diameter than the pure CoO nanowire without coating. In addition, it can be seen from Fig. 3d that ZIF-67 coating layer without obvious lattice fringe, indicating its low crystallinity, which is consistent with the characterization result of XRD. The TEM image in Fig. 3e shows that the original ordered 1D nanostructures of CoO@N-C remain well after annealing. However, in comparion with CoO@ZIF-67 nanowire, CoO@N-C exhibits rougher surface and nanopore composed of many nanoparticles, which may be due to gas release and further collapse of intermediate ZIF-67 product during the pyrolysis process. As shown in the high resolution TEM (HRTEM) image (Fig. 3f), the highly crystalline CoO nanoparticles and amorphous carbon substrate were produced after pyrolysis, and the CoO nanoparticles were encapsulated in several nanometers porous conductive carbon matrices, leaving massive mesopores for ion access and transportation (also see Fig. S3). Compared with other carbon coating methods, robust adhesion is generated between nitrogen-doped carbon and CoO nanoparticles, thus ensuring faster charge transfer on the electrode^25^. As a consequence, the nitrogen-doped carbon matrix could improve the electrical conductivity and accommodate a huge volume expansion/contraction of CoO nanoparticles, maintaining the integrity of the entire electrode. In addition, the nitrogen doped carbon produced by pyrolysis of MOFs is essentially a good conductive networks for both lithium-ion and electrons due to its interconnected nano-channels^26^.

**Figure 4:**
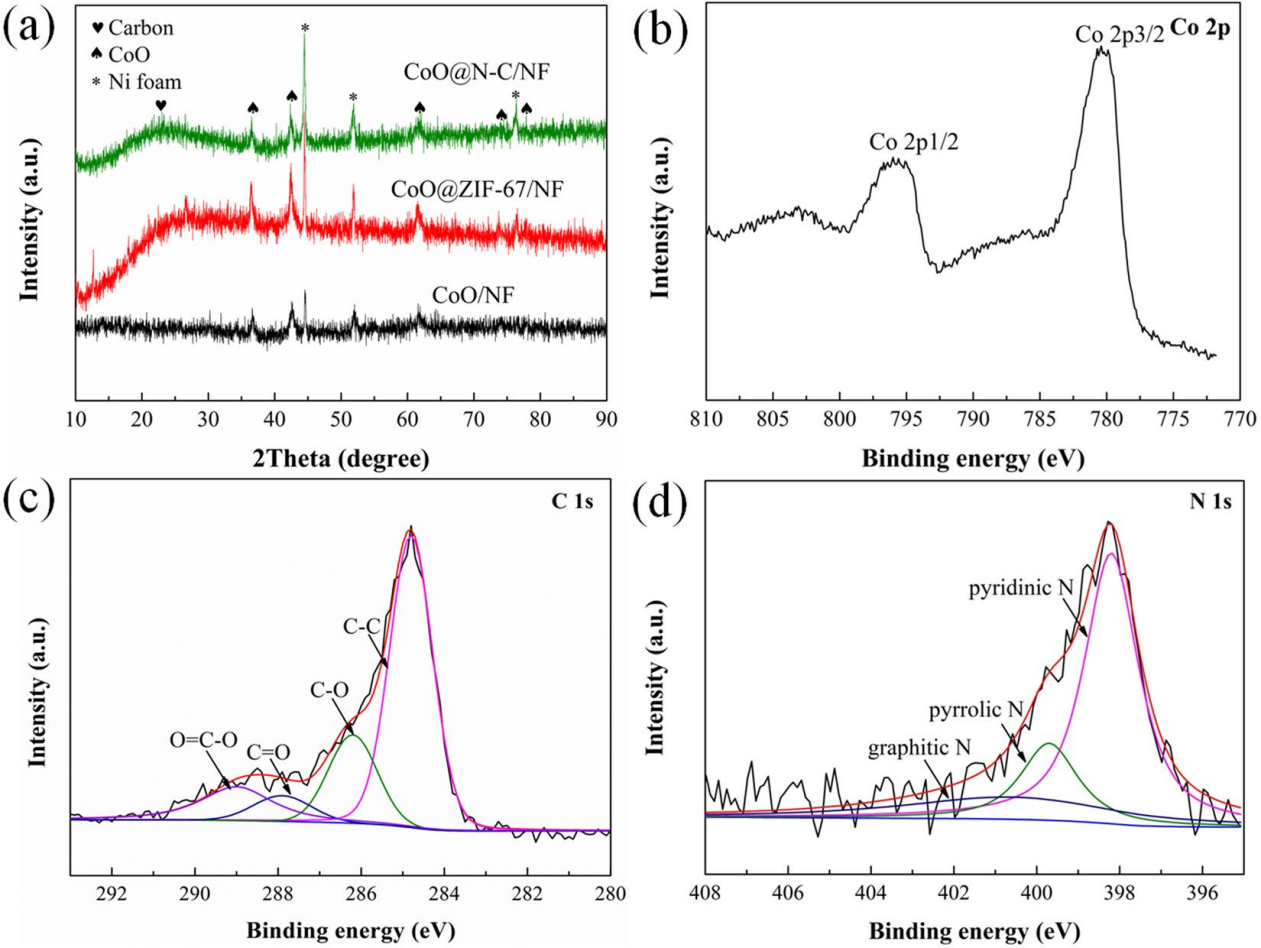
(**a**) X-ray diffraction patterns of as-prepared CoO/NF, CoO@ZIF-67/NF and CoO@N-C/NF. XPS spectra of the as-prepared CoO@N-C: (**b**) Co, (**c**) C and (**d**) N spectrum.

The crystal structure and phase purity of the product are confirmed by the XRD pattern. As the Fig. 4a shows, all the diffraction peaks of CoO in the CoO/NF, CoO@ZIF-67/NF and CoO@N-C/NF samples could be well linked to a pure phase of CoO with a cubic structure (JCPDS No. 48–1719), apart from the peaks originating from the Ni foam (marked by*)^27^. The middle X-ray diffraction (XRD) pattern of the ZIF-67 is weak on account of the strong diffraction originating from the Ni foam. In order to further prove that ZIF-67 was prepared after the reaction between CoO and Hmim, the XRD pattern (Fig. S4a) of ZIF-67 was matched well with the typical crystalline structure of ZIF-67 powder synthesized according to the mature method. In addition, CoO@N-C/NF sample with an additional wide peak between 2θ = 15–28°, corresponding to the carbon diffraction peak^28^. The presence of carbon in CoO@N-C samples is also confirmed by the full XPS spectrum.

The complete XPS analysis in Fig. S4b validates the existence of Co, O, C, and N, and their atomic ratios are 69.51%, 23.69%, 5.42%, and 1.39%, respectively. In addition, the XPS spectrum Co 2p (Fig. 4b) exhibits two small satellite peaks located at 780.8 eV and 796.6 eV, corresponding to Co 2p_3/2_ and Co 2p_1/2_,respectively, which can prove the formation of the CoO^29^. As shown in Fig. 4c, the XPS spectrum of C 1 s contains four peaks. A strong peak of all at 284.8 eV that correspond to the nonoxygenated graphitic carbon, and the binding energy values of other three weaker peaks are 286.2 eV, 287.9 eV and 289.0 eV, corresponding to the oxygenated carbons, carbonyl C, and carboxylate C, respectively^30^. The N 1 s spectrum in Fig. 4d can be decomposed into three peaks located at 398.4, 399.8, and 400.7 eV, corresponding to pyridinic, pyrrolic and graphitic N, respectively, which are bonded to the C atoms in CoO@N-C nanoparticles^31–33^.

**Figure 5:**
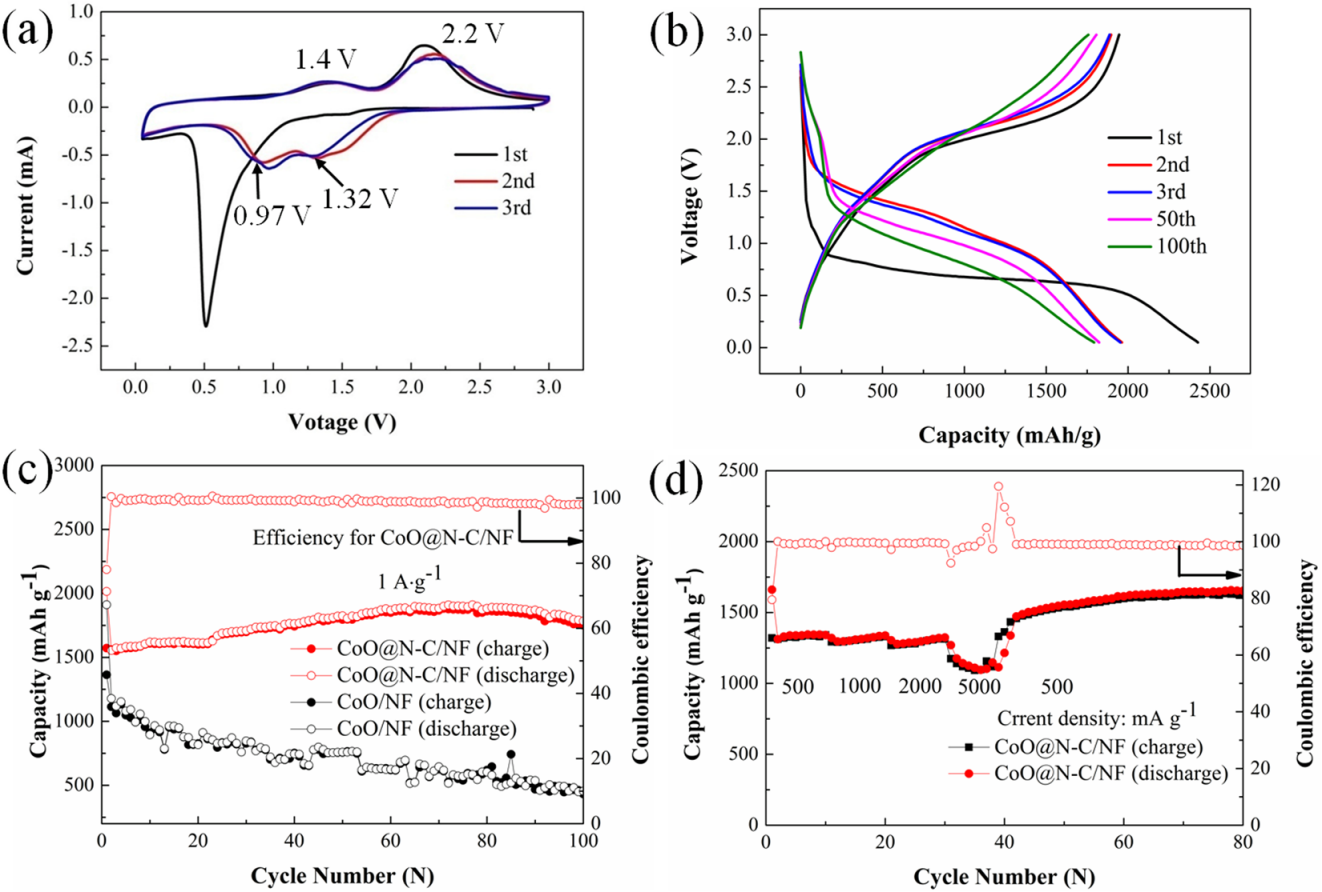
(**a**) The first three CV curves for CoO@N-C/NF; (**b**) The charge-discharge curves of hierarchical CoO@N-C/NF at a current density of 1 A g^−1^. (**c**) Cycle-life performance of CoO/NF and CoO@N-C/NF at a current density of 1 A g^−1^ over 100 cycles. (**d**) Rate performance and coulombic efficiency of CoO@N-C/NF.

In this work, CoO@N-C/NF were studied as anode material in LIBs. Figure 5a shows the initial three cycles CV curves of the CoO@N-C/NF electrode in the potential range of 0.05–3.0 V at a scan rate of 0.1 mV s^−1^. During the first cathodic scan, one strong reduction peak between 1.0 and 0.37 V can be attributed to the reduction of Co^2+^ to metallic Co during the insertion of Li^+^, lithium ion insertion into the carbon and also formation of the solid electrolyte interface (SEI) film^29^. In the following cathode scanning, the cathodic peaks shift to higher voltages at 1.32 and 0.97 V, which is attributed to the reduction of CoO to metallic Co and the formation of SEI film resulting from the electrochemically driven electrolyte reduction^30,34,35^. In anodic scans, the peak at ≈1.4 V can be attributed to the partial decomposition of SEI film and the extraction of Li^+^ from carbon^36,37^. The anodic peak at ≈2.2 V is related to the electrochemical reaction between Li_2_O and Co (Li_2_O + Co → CoO + 2Li)^37,38^. In the following cycles, the good overlap of the CV curves indicates the highly reversible of the electrochemical reactions.

The core-shell CoO@N-C with a conductive carbon encapsulated CoO nanoparticles are expected to show excellent lithium storage properties which was indeed confirmed by the experimental results. Figure 5b shows the discharge/charge profiles of CoO@N-C/NF at a current density of 1 A g^−1^ in the 1st, 2nd, 3rd, 50th, 100th cycle. In the first discharge, the long stable voltage stage can be clearly observed at 0.75 V, which matched well with the above CV results (Fig. 5a). The first discharge and charge capacity reach up to 2015.2 and 1572.8 mAh g^−1^, respectively, corresponding to a initial Coulombic efficiency of 78.04%. The initial capacity loss is due to the incomplete conversion reaction and the formation of the SEI film. It should be emphasized that discharge-charge curves for the 50th and 100th almost no obvious changes are observed, indicating good cycling stability.

Figure 5c compares the cycle performance of the CoO@N-C/NF and CoO/NF electrodes at 1 A g^−1^. After 100 cycles, the CoO@N-C/NF electrode provides a high reversible capacity of 1884.1 mAh g^−1^ much higher than that (454.6 mAh g^−1^) of the pure CoO/NF anode and larger than the theoretical value (716 mAh g^−1^). The phenomenon of over-theoretical capacity is similar to the interesting results of other transition metal oxides^9,39–41^. The extra capacities could be affiliated with the reversible growth of polymeric gels originating from the electrolyte decomposition and the further lithium storage via interfacial charging at the metal/Li_2_O interface^36,42,43^. Moreover, there is a trend of the capacity of CoO@N-C/NF increase gradually, while the capacity of CoO/NF does not increase in Fig. 4c. For the CoO/NF electrode, just as most transition metal oxides, the pure CoO nanowire arrays without the nitrogen-doped carbon coating exhibit rapid capacity fading, which is due to their large volume change causing the pulverization of the electrode material during the charge/discharge process. In contrast, the CoO@N-C/NF electrode with an upward trend in capacity is attributed to the synergistic effect between CoO nanoparticles components and nitrogen-doped carbon skeleton. The nitrogen doped carbon coating plays a crucial role in improving the electronic conductivity, buffering the volume change, and enhancing the stability of the electrode. At the same time, the reversible growth of a polymeric gel-like film resulting from the kinetically activated electrolyte degradation have been suggested as one of the possible reasons for the capacity increase. Of course, in addition to the positive factors causing the capacity rise, the large volume change during the charge/discharge process results in the pulverization of the electrode and the capacity decreases correspondingly. These two factors competitively affect the trend of capacity retention. Similar phenomena have also been observed in other material systems^44–46^. For many energy storage devices, rate capability is one of the critical factor in practical application. Figure 5d futher shows the rate capabilities performance of CoO@N-C/NF electrode for current rates from 500 to 5000 mA g^−1^ for each 10 cycles, corresponding to the discharge of 1369.2, 1315.2, 1290, and 1169.2 mAh g^−1^, respectively. What is more important, when the rate is back to 0.5 A g^−1^, the reversible specific capacity of the samples recover to their initial values, implying a very stable cycling performance. In addition, when the current density is different, the charge-discharge curve does not have a large voltage hysteresis change. Their similarity means that only limited polarization exists in this system, even in the case of large current densities, thus indicating an excellent rate capability (Fig. S5)^24^. To our knowledge, this is the highest capacity with similar electric current density that has been reported for CoO-based materials, and the comparative results are listed in Supporting Information, Table S1.

The improvement of CoO@N-C/NF electrochemical performance might be ascribed to their unique structural characteristics as the following aspects. Firstly, the vertical core-shell architecture nanowires array was directly attached to the Ni foam, avoiding the addition of conductive carbon and binders, and greatly reducing the “dead volume” in the electrode, which will facilitate charge transport. Secondly, the continuous, complete and uniform nitrogen-doped carbon skeleton can effectively withstand the volume change in the process of alloying-dealloying, thus alleviating the pulverization problem to a certain extent and improving the cycling stability. To prove this, we compared the SEM of the CoO/NF and CoO@N-C/NF electrodes after 100 cycles of charge and discharge (see Fig. S6 in the Supporting Information). As expected, the nanoarrays morphology of CoO@N-C/NF electrode was basically retained after 100 cycles. In contrast, the pulverization of the CoO/NF electrode was observed due to the aforementioned volume excursion effects. Finally, there are many competitive advantages to the 1D robust nanowires arrays, such as more active sites, short ion diffusion distance, superior electron collection efficiency, and even attractive synergetic properties.

**Figure 6:**
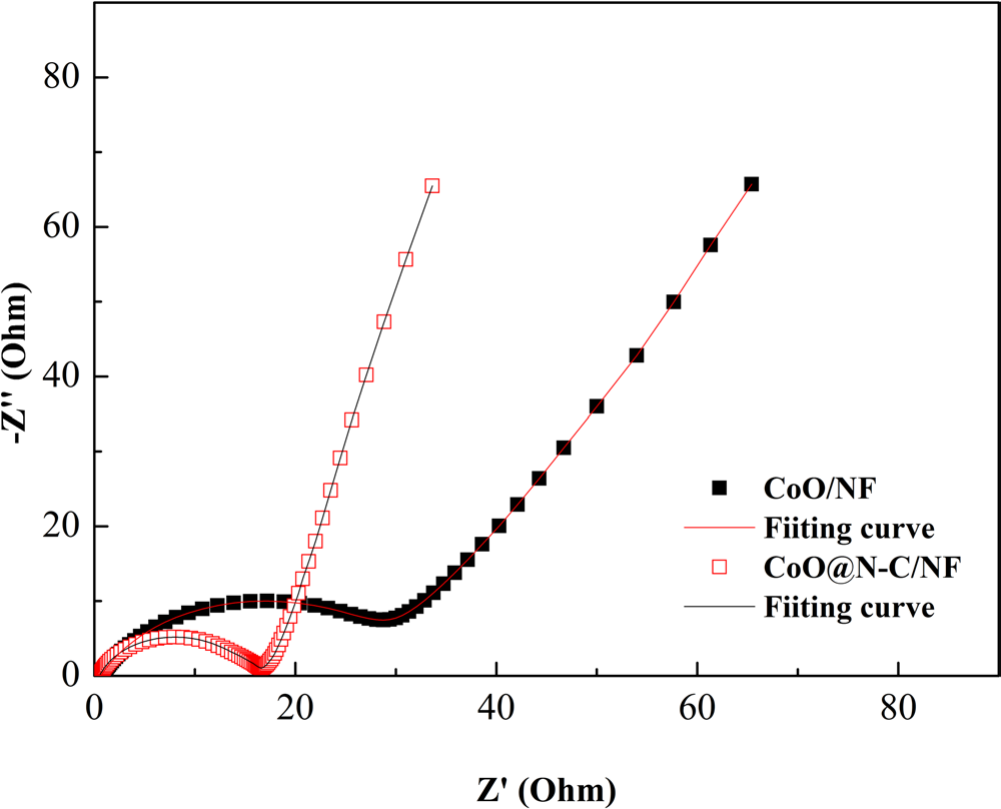
The Nyquist plots of pure CoO/NF and CoO@N-C/NF electrodes.

In order to further understand the reason why CoO@N-C/NF shows the best capacity than pure CoO/NF, the electrochemical impedance spectroscopy (EIS) measurements were carried out within the frequency range of 0.01–100 kHz. Figure 6 displays the impedance plots together with the equivalent circuit model of CoO@N-C/NF and pure CoO/NF electrodes after the 3th cycle at 1 A g^−1^. The Nyquist plots consists of one depressed semicircle in the high frequency and a sloped line at low frequency. In general, the semicircle is related to the internal resistance (R_e_) of the battery, the resistance (R_f_) and constant phase element (CPE_f_) of SEI film, the charge transfer resistance (R_ct_) and constant phase element (CPE_ct_) of the electrode/electrolyte interface. The sloped line indicates that the Warburg impedance (Z_w)_ is attributed to the diffusion of Li^+^. The SEI film resistance R_f_ and charge-transfer resistance R_ct_ of the CoO@N-C/NF electrode are 5.2 Ω and 9.8 Ω, which are much less than the corresponding value of the pristine CoO/NF electrode (7.4 Ω and 17.43 Ω). It is obvious that the special N-C matrix structure can significantly enhance the electronic conductivity of the materials surface, which is beneficial to the rapid transport of electrons within the hollow CoO nanoparticles during electrochemical lithium insertion/extraction process, thus greatly improving to the electrochemical performance of CoO@N-C/NF electrode.

## Conclusions

In summary, we have successively synthesized the unique hierarchical CoO@N-C nanowires on 3D conductive Ni foam substrates based on a facile and extensible *in-situ* ion exchange process. Excitingly, CoO@N-C/NF electrode demonstrates excellent electrochemical performance compared to the precursor of CoO/NF, benefiting to the sufficient conductivity, unique hierarchical microstructure and good structure stability of the nanowires arrays. CoO@N-C/NF electrode exhibits a high reversible capacity of 1884.1 mAh g^−1^ at 1 A g^−1^ after 100 cycles and outstanding rate capability. Our results indicate that the CoO@N-C/NF electrodes without any auxiliary materials are expected to open up new opportunities for CoO-based material to power electronic devices.

## Methods

### Preparation of CoO/NF

Synthesis of CoO nanowire arrays on Ni foam (CoO/NF). All the chemicals are directly used after purchase without further purification. The hierarchical CoO nanowire arrays was synthesized through a modified two step of hydrothermal and annealing^47^. Typically, 2 mmol of Co(NO_3_)_2_.6H_2_O, 10 mmol of CO(NH_2_)_2_, and 8 mmol of NH_4_F were dissolved in 36 mL of deionized water under stirring. The resultant solution was added to a 100 mL Teflon-lined autoclave immersing a piece of cleaned Ni foam. The synthesis temperature was maintained at 120 °C for 10 h and after the equipment cooled down to room temperature naturally. The Ni foam with the precursor was taken out and rinsed with deionized water for several times. The CoO/NF is finally obtained by annealing the above precusor at 450 °C in Ar gas for 4 h at a ramping rate of 2 °C/min.

### Preparation of CoO@ZIF-67/NF

In detail, 40 mmol of 2-methylimidazole (Hmim) was added into a beaker containing 10 mL each of deionized water and ethanol. Then, the rinsed intermediate of CoO/NF was immersed into the solution above and put it into the shaker to maintain a speed of 190 revolutions and maintain it at room temperature for 9 hours. The purple resultant product was fetched out and rinsed by ethanol for three times and then dried at 80 °C for 12 h to obtain CoO@ZIF-67/NF. For further investigate the formation process of CoO@ZIF-67/NF, the resultant products were fetched out at the reaction times of 5 min, 1 h, 6 h, and 12 h.

### Preparation of CoO@N-C/NF

The obtained CoO@ZIF-67/NF was annealed at 550 °C for 2 h in argon atmosphere, and then annealed in air at 300 °C for 1 h with a heating rate of 2 °C/min to obtain the CoO@N-C/NF nanocomposite. The mass loading of CoO@N-C was around 1.34 mg cm^−2^, as calculated using the following formula^48^:

$${\rm{m}}=({{\rm{m}}}_{2}-{{\rm{m}}}_{1})\cdot {{\rm{S}}}_{{\rm{Ni}}}/{\rm{S}}$$

where m is the weight of CoO@N-C; m_1_ is the weight of fresh Ni foam and m_2_ is the weight of Ni foam covered with active material. S_Ni_ is the surface area of the electrode that was used in the coin cell and S is the total surface area of Ni foam covered with the active material.

### Characterization techniques

The crystal structures and morphologies of the samples was characterized by X-ray diffractometer (XRD, Bruker AXS D8), scanning electron microscope (SEM, JEOL JSM-6490LV) and transmission electron microscope (TEM, FEI Tecnai G2). Surface analysis of the samples was examined using X-ray photoelectron spectroscopic (XPS, PHI1600 ESCA).

### Electrochemical measurements

The electrochemical characterization were carried out using CR-2032 coin cells. The fabricated CoO@N-C/NF was used as working electrode and lithium metal as the counter electrode. The separator membrane was Celgard 2320. A electrolyte solution of 1 M LiPF_6_ consists of EC, DMC and EMC (v/v/v = 1: 1: 1). Galvanostatic charge–discharge was carried out on a LAND battery tester (LAND CT 2001A, China) in the fixed voltage window of 0.05–3.0 V. Cyclic voltammetry (CV) and electrochemical impedance spectroscopy (EIS) were measured on a CHI604 C electrochemical workstation. The impedance spectra were carried out in the frequency range of 100 kHz to 0.01 Hz.

## Supplementary information

N-doped Carbon Coated CoO Nanowire Arrays Derived from Zeolitic Imidazolate Framework-67 as Binder-free Anodes for High-performance Lithium Storage
