# Supplementary material for: N-doped Carbon Coated CoO Nanowire Arrays Derived from Zeolitic Imidazolate Framework-67 as Binder-free Anodes for High-performance Lithium Storage
Source: Sci Rep. 2019 Apr 11;9:5934. doi: 10.1038/s41598-019-42371-y (PMC6459822; doi:10.1038/s41598-019-42371-y)
Supplement: Supplementary file 1 — N-doped Carbon Coated CoO Nanowire Arrays Derived from Zeolitic Imidazolate Framework-67 as Binder-free Anodes for High-performance Lithium Storage [file 41598_2019_42371_MOESM1_ESM.docx]

Supporting Information:

**N-doped Carbon Coated CoO Nanowire Arrays Derived from Zeolitic Imidazolate Framework-67 as Binder-free Anodes for**

**High-performance Lithium Storage**

**Dongxia Wang^a^, Bo Yan^a^, Yujuan Guo^a^, Long Chen^a^, Feng Yu^a^*, Gang Wang^a,b^***

^a^ *School of Chemistry and Chemical Engineering, Key Laboratory for Green Processing of Chemical Engineering of Xinjiang Bingtuan, Shihezi University, Shihezi 832003, China*

^b^ *Key Laboratory of Materials-Oriented Chemical Engineering of Xinjiang Uygur Autonomous Region, Shihezi 832003, China*


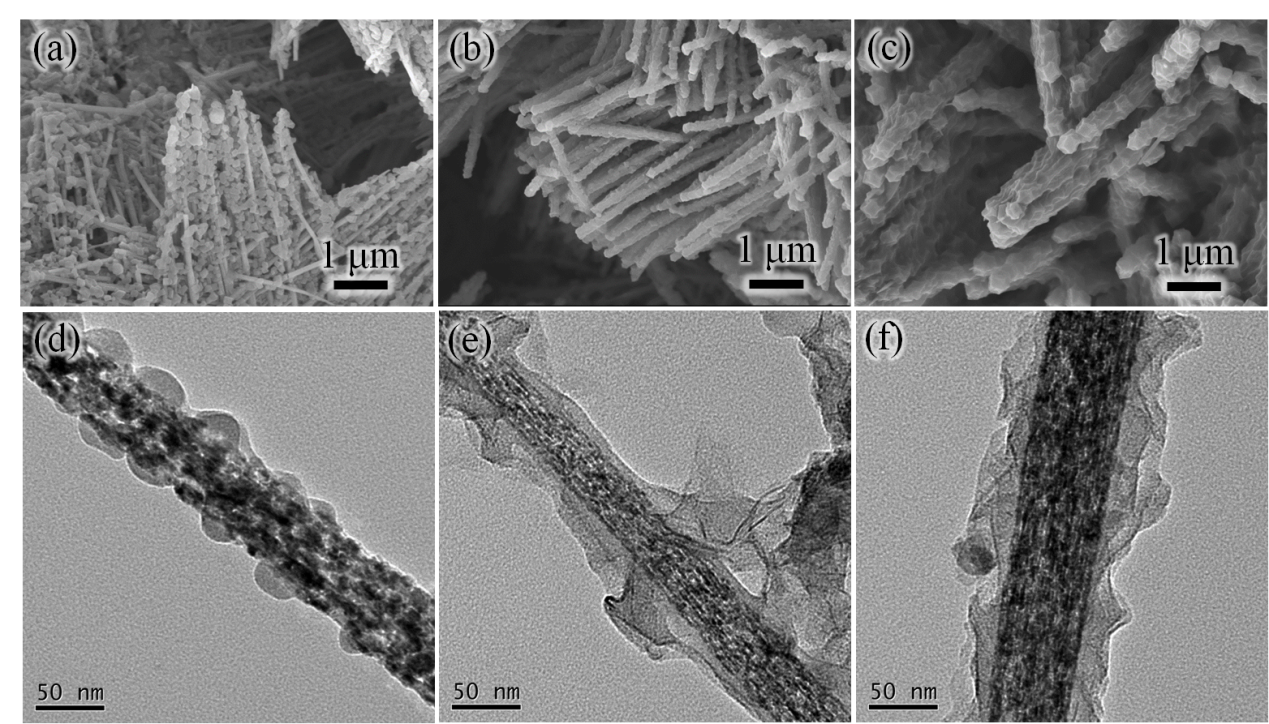


Figure.S1 SEM and TEM images of Ni@CoO@ZIF-67 at different reaction time lengths. (a-c) SEM and (d-f) TEM images of Ni@CoO@ZIF-67 prepared at different reaction time length: 5 min, 1 h, and 6 h, respectively. Inset in (a-c): the illustration corresponding to evolution process.


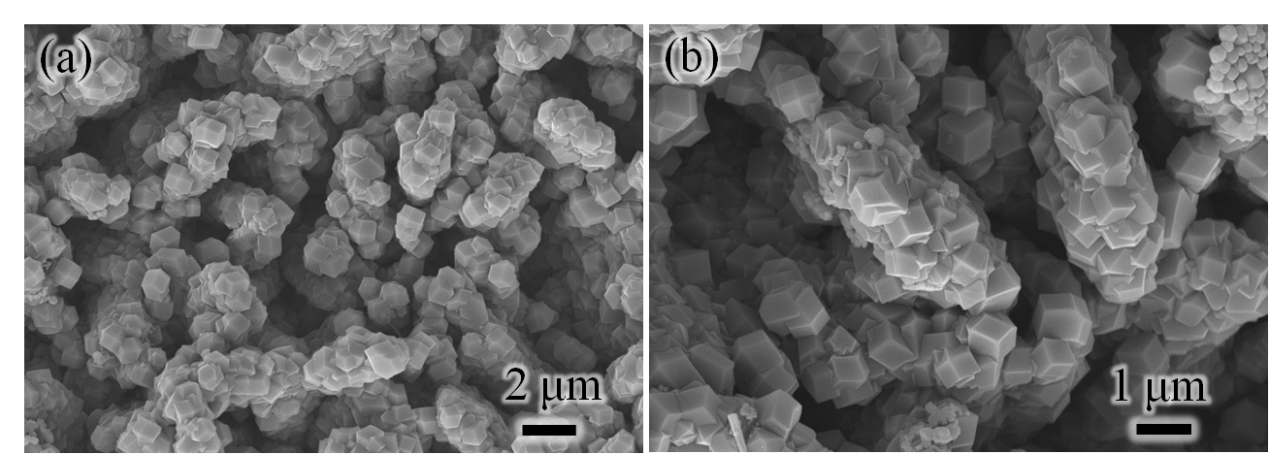


Figure.S2 SEM images of Ni@CoO@ZIF-67 after the chemical transformation with 2-methylimidazole for 12h.


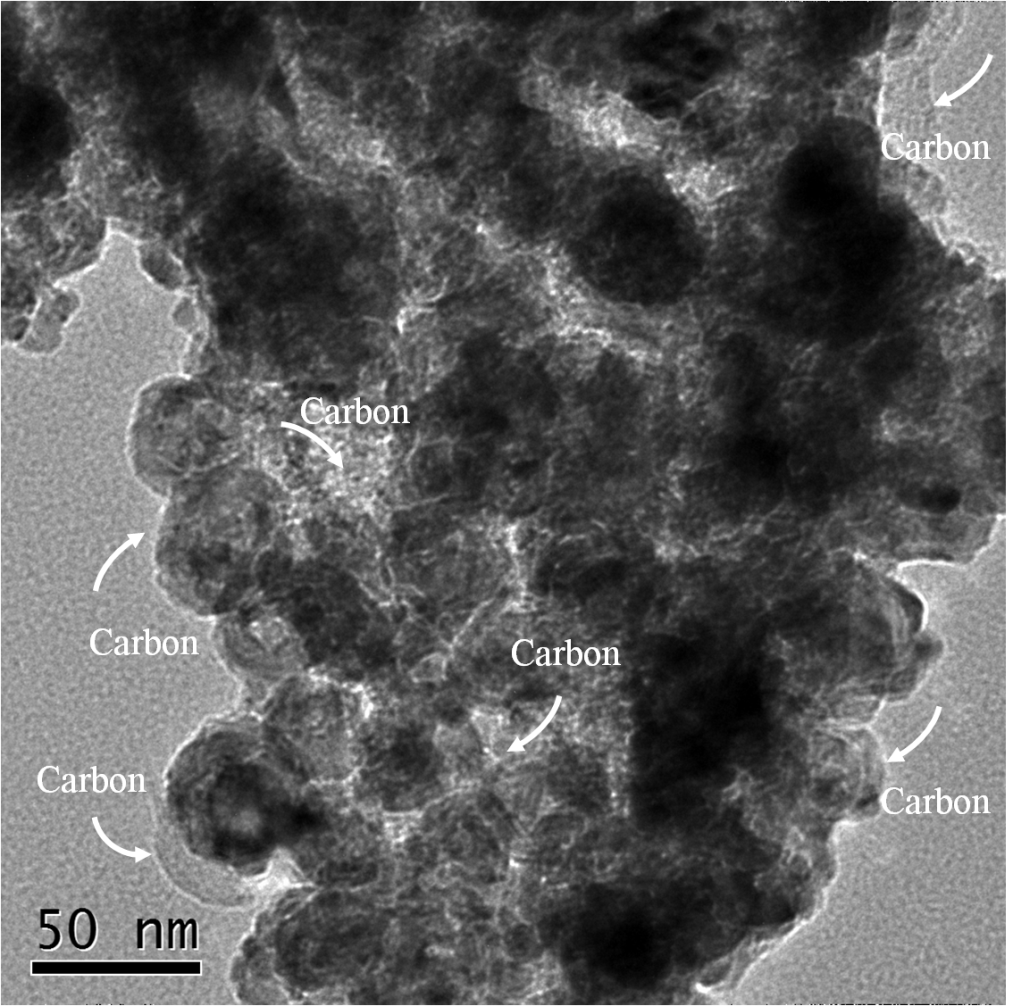


Figure.S3 High-magnification TEM image of core–shell CoO@N-C/NF.


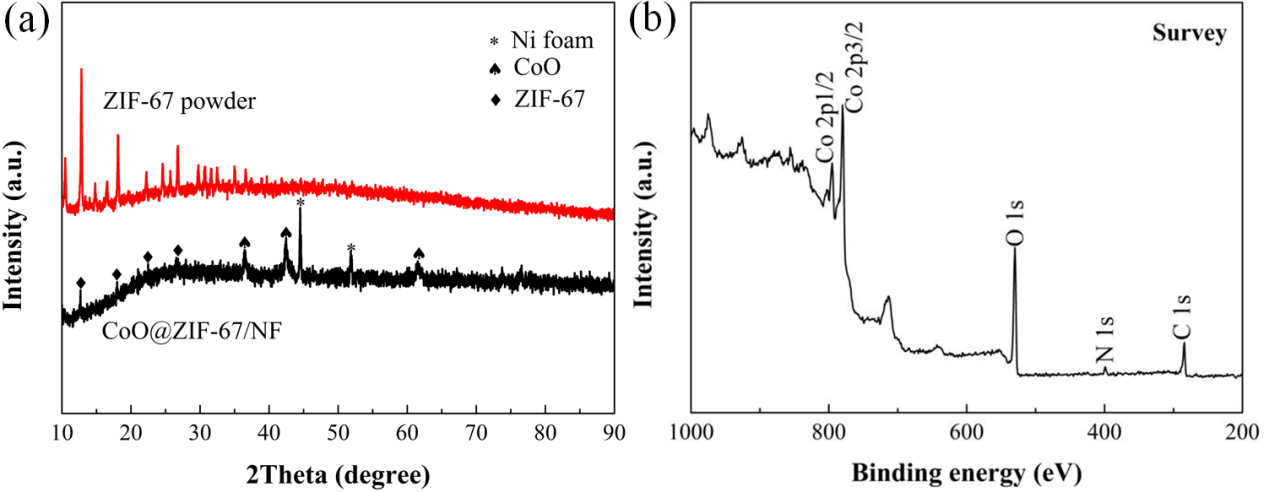


Figure. S4 (a) XRD patterns of ZIF-67 powder and CoO@ZIF-67/ NF. (b)survey spectrum XPS spectra of the as-prepared CoO@N-C.





Figure. S5 The charge–discharge curves of hierarchical CoO@N-C/NF at different current densities.


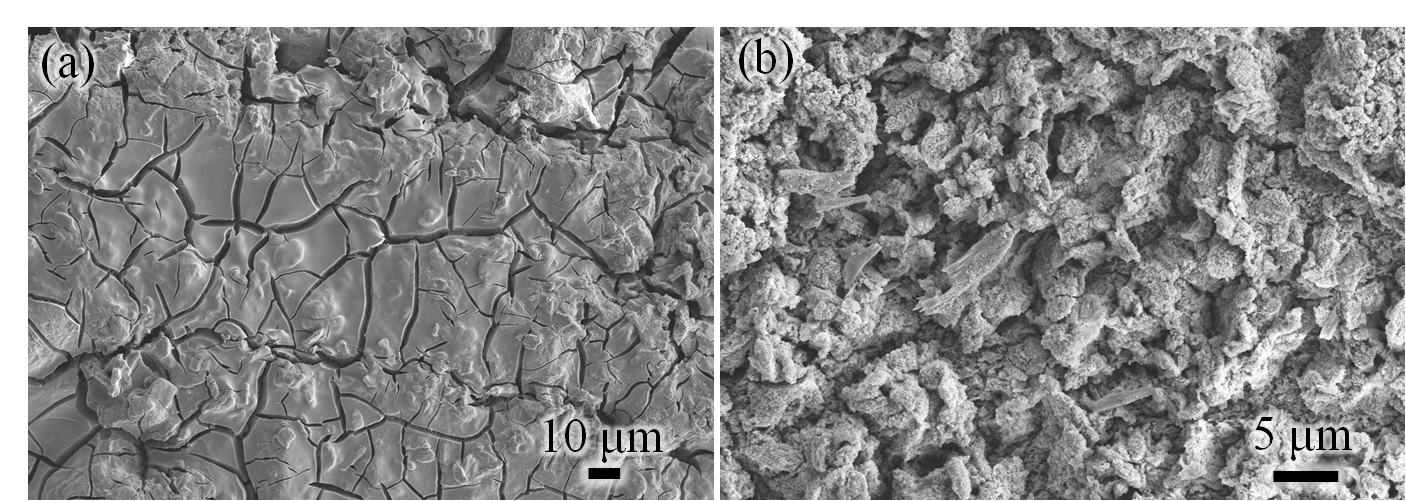


Figure.S6 SEM images of (a) CoO/NF and (b) CoO@N-C/NF after 100 cycles at a current density of 1000mA g^-1^.

Table S1. Specific capacities and cycling properties of the CoO based materials as anodes for LIBs

| Electrode materials | Morphology | Reversible Capacity | Current density | Ref. |
| --- | --- | --- | --- | --- |
| CoO@graphene | Quantum Dot/  Nanosheet | 1592 mAh g^-1^ (50th) | 50 mA g^-1^ | 1 |
| CoO@graphene | Hybrid Nanosheets | 1018 mAh g^-1^ (50th) | 200 mA g^-1^ | 2 |
| CoO/N-C | nanoparticle cookies | 1383 mAh g^- 1^ (200th) | 100 mA g^-1^ | 3 |
| CoO@N-C | Nanocubes | 598.3 mAh g^-1^ (50th) | 100 mA g^-1^ | 4 |
| CoO@N,S-codoped carbon | Foam | 887 mAh g^-1^ (200th) | 1000 mA g^-1^ | 5 |
| CoO nanonets | Nanonet | 637 mAh g^-1^ (200th) | 100 mA g^-1^ | 6 |
| CoO/C | spherical | 855 mAh g^-1^ (100th) | 1400 mA g^-1^ | 7 |
| CoO@NC | microspheres | 1244 mAh g^- 1^ (120th) | 89 mA g^-1^ | 8 |
| CoO NWCs on  copper foil | nanowires | 1248.8 mAh g^-1^ (50th) | 716 mA g^-1^ | 9 |
| CoO@BNG | nanotubes | 1485 mAh g^-1^ (20th) | 100 mA g^-1^ | 10 |
| porous CoO | microsphere | 950 mAh g^-1^ (120th) | 200 mA g^-1^ | 11 |
| hierarchical CoO | microflower | 1297.9 mAh g^-1^(500th) | 454.5 mA g^-1^ | 12 |
| C@CoO | porous spheres | 669 mAh g^- 1^ (400th) | 500 mA g^-1^ | 13 |
| CoO@N-C | Nanowires composed of nanoparticles | 1884.1 mAh g^-1^(100th) | 1000 mA g^-1^ | This work |

**References**:

[1] C. Peng, B. Chen, Y. Qin, S. Yang, C. Li, Y. Zuo, S. Liu, J. Yang, Facile ultrasonic synthesis of CoO quantum dot/graphene nanosheet composites with high lithium storage capacity, Acs Nano, 6 (2012) 1074–1081.

[2] Y. Sun, X. Hu, W. Luo, Y. Huang, Ultrathin CoO/Graphene Hybrid Nanosheets: A Highly Stable Anode Material for Lithium-Ion Batteries, The Journal of Physical Chemistry C, 116 (2012) 20794-20799.

[3] S. Wang, M. Chen, Y. Xie, Y. Fan, D. Wang, J.J. Jiang, Y. Li, H. Grützmacher, C.Y. Su, Nanoparticle Cookies Derived from Metal-Organic Frameworks: Controlled Synthesis and Application in Anode Materials for Lithium-Ion Batteries, Small, 12 (2016) 2365-2375.

[4] K. Xie, W. Ping, Y. Zhou, Y. Ye, W. Hui, Y. Tang, Y. Zhou, T. Lu, Nitrogen-Doped Carbon-Wrapped Porous Single-Crystalline CoO Nanocubes for High-Performance Lithium Storage, Acs Appl Mater Interfaces, 6 (2014) 10602-10607.

[5] F. Wang, H.Y. Zhuo, X.G. Han, W. Chen, D. Sun, Foam-like CoO@N,S-codoped carbon composition derived from a well-designed N,S-rich Co-MOF for lithium-ion battery, Journal of Materials Chemistry A, 5 (2017) 22964-22969.

[6] X. Zhou, Y. Zhong, M. Yang, Q. Zhang, J. Wei, Z. Zhou, Co_2_(OH)_2_CO_3_ Nanosheets and CoO Nanonets with Tailored Pore Sizes as Anodes for Lithium Ion Batteries, Acs Applied Materials & Interfaces, 7 (2015) 12022–12029.

[7] J.H. Kim, Y.C. Kang, Electrochemical properties of micron-sized, spherical, meso- and macro-porous Co_3_O_4_ and CoO-carbon composite powders prepared by a two-step spray drying process, Nanoscale, 6 (2014) 4789-4795.

[8] G. Liu, J. Shao, Pomegranate-like CoO@nitrogen-doped carbon microspheres with outstanding rate behavior and stability for lithium storage, Journal of Materials Chemistry A, 5 (2017) 9801-9806  .

[9] K. Cao, L. Jiao, Y. Liu, H. Liu, Y. Wang, H. Yuan, Ultra‐High Capacity Lithium‐Ion Batteries with Hierarchical CoO Nanowire Clusters as Binder Free Electrodes, Advanced Functional Materials, 25 (2015) 1082-1089.

[10] H. Tabassum, R. Zou, A. Mahmood, Z. Liang, Q. Wang, H. Zhang, S. Gao, C. Qu, W. Guo, S. Guo, A Universal Strategy for Hollow Metal Oxide Nanoparticles Encapsulated into B/N Co‐Doped Graphitic Nanotubes as High‐Performance Lithium‐Ion Battery Anodes, Advanced Materials, 30 (2018) 1705441.

[11] L. Chang, K. Wang, L. Huang, Z. He, H. Shao, J. Wang, Hierarchically porous CoO microsphere films with enhanced lithium/sodium storage properties, Journal of Alloys & Compounds, 725 (2017) 824-834.

[12] L. Chang, K. Wang, L. Huang, Z. He, S. Zhu, M. Chen, H. Shao, J. Wang, Hierarchical CoO microflower film with excellent electrochemical lithium/sodium storage performance, Journal of Materials Chemistry A, 5 (2017) 20892-20902.

[13] X. Tang, Q. Feng, J. Huang, K. Liu, X. Luo, Q. Peng, Carbon-coated cobalt oxide porous spheres with improved kinetics and good structural stability for long-life lithium-ion batteries, Journal of Colloid & Interface Science, 510 (2017) 368-375.
